# Dentin Matrix Protein 1 Silencing Inhibits Phosphorus Utilization in Primary Cultured Tibial Osteoblasts of Broiler Chicks

**DOI:** 10.3389/fvets.2022.875140

**Published:** 2022-04-26

**Authors:** Tingting Li, Yanqiang Geng, Yun Hu, Liyang Zhang, Xiaoyan Cui, Weiyun Zhang, Feiyu Gao, Zongping Liu, Xugang Luo

**Affiliations:** ^1^Poultry Mineral Nutrition Laboratory, College of Animal Science and Technology, Yangzhou University, Yangzhou, China; ^2^Mineral Nutrition Research Division, Institute of Animal Science, Chinese Academy of Agricultural Sciences, Beijing, China; ^3^College of Veterinary Medicine, Yangzhou University, Yangzhou, China

**Keywords:** broiler, DMP1, phosphorus, utilization, primary tibial osteoblast

## Abstract

Three experiments were carried out in the present study to investigate whether dentin matrix protein 1 (DMP1) was involved in regulating phosphorus (P) metabolic utilization in primary cultured tibial osteoblasts of broiler chicks. Experiment 1 was conducted to select the optimal osteogenic inductive culture medium and the optimal induction time in primary cultured tibial osteoblasts of broiler chicks. In experiment 2, the siRNAs against DMP1 were designed, synthesized and transfected into primary cultured tibial osteoblasts of broiler chicks, and then the inhibitory efficiencies of siRNAs against DMP1 were determined, and the most efficacious siRNA was selected to be used for the DMP1 silencing. In experiment 3, with or without siRNA against DMP1, primary cultured tibial osteoblasts of broiler chicks were treated with the medium supplemented with 0.0, 1.0 or 2.0 mmol/L of P as NaH_2_PO_4_ for 12 days. The P metabolic utilization-related parameters were measured. The results showed that the osteogenic induced medium 2 and 12 days of the optimal induction time were selected; Among the designed siRNAs, the si340 was the most effective (*P* < 0.05) in inhibiting the DMP1 expression; DMP1 silencing decreased (*P* < 0.05) the expressions of *DMP1* mRNA and protein, P retention rate, mineralization formation, alkaline phosphatase activity and bone gla-protein content in tibial osteoblasts at all of added P levels. It is concluded that DMP1 silencing inhibited P utilization, and thus DMP1 was involved in regulating P metabolic utilization in primary cultured tibial osteoblasts of broiler chicks, which provides a novel insight into the regulation of the P utilization in the bone of broilers, and will contribute to develop feasible strategies to improve the bone P utilization efficiency of broilers so as to decrease its excretion.

## Introduction

Phosphorus (P) supply plays a critical role in the bone development and mineralization of broiler chickens (1–4) since a high proportion of P was deposited in bone as the component of hydroxyapatite (5). However, early fast growth rate of broilers is generally accompanied by a high incidence of leg problems as the bone development cannot keep pace with such a fast rate of increase in body weight (6). Approximately 80% of P absorbed from the small intestine is deposited in the bone as hydroxyapatite (5), and this deposition process is mainly carried out by osteoblasts (7). Furthermore, the uptake of P by osteoblasts is usually considered as a major factor regulating the bone development, mineralization and remodeling (8). Studies on the above aspects would provide a novel insight into the regulation of the P utilization and its mechanisms in the bone development of broilers and also develop the feasible strategies to improve the bone P utilization so as to decrease its excretion into the environment.

The major function of osteoblasts is to produce the organic constituents of the bone extracellular matrix that facilitate its mineralization by inorganic compounds (8, 9). Osteoblastic mineralization is an important osteoplastic link and specific biological process of the bone in which calcium (Ca) and P deposit on type-I collagen to form hydroxyapatite. Besides being a component of hydroxyapatite, inorganic phosphate acts as an intracellular signaling molecule to regulate the osteoblastic mineralization (10). Therefore, primary cultured osteoblasts may be an ideal *in vitro* model for exploring the patterns and mechanisms of the bone P utilization in broilers. It was reported that osteoblasts could be isolated from the calvarias, iliac cancellous bone, cervical bone, and femurs of humans, rats, mice, and rabbits using a sequential enzymatic digestion method, explant method or the combined method of the above two methods (11–13). Recently, we have successfully established the primary cultured tibial osteoblast model of broilers by the explant method (14), which provided an attractive means for the study of the effect of dentin matrix protein 1 (DMP1) silencing on P utilization in osteoblasts of broilers.

The DMP1 is a multi-functional protein that has been found to play a significant role in biomineralization (15). The DMP1 was firstly cloned in bone-like mineralized tissues, including dentin, bone, cartilage or cementum of human, mouse, and chicken (16–18). As a phosphate regulator, DMP1 is responsible for the bone mineralization and phosphate homeostasis in osteocytes (19). Study of fetal rat calvaria cell cultures showed that the expression of DMP1 is associated closely with the bone nodule formation and mineralization (20). The DMP1-null mice exhibited poor skeletal mineralization and rickets combined with low serum phosphate and elevated serum fibroblastic growth factor 23 (FGF23) (19, 21). Overexpression of DMP1 in Nestin^+^ cells leaded to decreased bone mass (22). Our previous *in vivo* studies have indicated that the bone P utilization and bone development might be regulated by related hormones, local-derived regulators as well as BMP and mitogen-activated protein kinase signing pathways in the tibia of broiler chicks (1, 23). The DMP1 has been found to be involved in regulating P homeostasis and bone mineralization in mammals (24, 25). Latest studies *in vivo* and *in vitro* suggested that bone P retention or osteoblastic P utilization might be regulated by DMP1 (1, 26), implying a possible contribution of DMP1 to the bone P utilization of broilers. However, whether DMP1 is involved in regulating P metabolic utilization in primary cultured tibial osteoblasts of broiler chicks has not been reported before. Therefore, we hypothesized that DMP1 silencing would inhibit P utilization, and thus DMP1 would be involved in regulating P metabolic utilization in primary cultured tibial osteoblasts of broiler chicks. To test the above hypothesis, the objective of the current study was to investigate the effect of the gene silencing of DMP1 by using siRNAs on P utilization and related parameters in primary cultured tibial osteoblasts of broiler chicks.

## Materials and Methods

The study protocol was approved by the Animal Care Committee of the Department of Animal Science and Technology of Yangzhou University, Yangzhou, China [permit number: SYXK (Su) 2017-0027] and conducted in accordance with the guidelines of the Animal Use Committee of the Chinese Ministry of Agriculture (Beijing, China). All efforts were made to minimize animal suffering.

### Isolation and Cultivation of Primary Tibial Osteoblasts of Broiler Chicks

The primary tibial osteoblasts of broiler chicks were isolated and cultivated by using an approach of the previous method reported in broiler chicks (14). Tibial bones from both legs were obtained from 1-day-old commercial chicks (male Arbor Acres broilers, purchased from Jinghai Broiler Livestock Industry Group, Haimen, China) after cervical dislocation. The birds were soaked in alcohol for 2 min after cervical dislocation. Legs were removed from hip joint and metacarpal. Dissected legs were kept in Dulbecco's Modified Eagle's medium (DMEM; Macgene, Beijing, China) containing 1% penicillin-streptomycin (Gibco, Grand Island, NY, USA) until connective tissues and muscles were completely removed. The epiphysis of the tibial bones was removed to expose the bone marrow cavity. Bone marrow inside the bone was flushed with Dulbecco's Phosphate Buffer Saline (D-PBS) supplemented with 1% penicillin-streptomycin. The tibial bones were split longitudinally with a scalpel and then scraped with a curved forceps to remove the hematopoietic cells adhered to the compact bones. The cleaned bone flakes were cut into 1 mm^2^ with sterile surgical scissors, plated in 60-mm cell culture dishes, and then incubated in the complete culture medium, consisting of DMEM, supplemented with 1% penicillin-streptomycin, 1% L-glutamine (Gibco, Grand Island, NY, USA) and 15% fetal bovine serum (FBS; Gibco, Auckland, New Zealand) at 37°C in a humidified atmosphere containing 5% CO_2_. Once the cells migrated from bone pieces and covered up to 80% of the flask bottom surface, the cells were washed twice with D-PBS (Gibco, Grand Island, NY, USA), dissociated with 0.25% Trypsin-EDTA (Macgene, Beijing, China) for 1 min and subcultured in 6-well plates coated with rat tail collagen at a density of 5 × 10^5^ cells/well in the above complete culture medium. This passage was marked as P1. The P1 cells were used for all subsequent experiments.

### Experimental Design and Treatments, Sample Collections, and Preparation

Experiment 1 was carried out to evaluate the effects of four osteogenic induced mediums (OIMs) on osteoblastic mineralization and select the optimal induction time in primary cultured tibial osteoblasts of broiler chicks. There were a total of 16 treatments involving a 4 (OIM type) × 4 (inducted time point) factorial arrangement in a completely randomized design. When the P1 cells grew to 80–90% confluence, complete culture medium was replaced with different OIMs. Four OIM types were control medium (CON, complete culture medium), the conventional OIM [OIM1, control medium supplemented with 100 nmmol/L dexamethasone (Dex), 50 μg/mL ascorbic acid (Asc), and 10 mmol/L β-sodium glycerophosphate (β-Gly)], OIM2 (control medium supplemented with 100 nmmol/L Dex and 50 μg/mL Asc) and OIM3 (OIM2 supplemented with 10 mmol/L NaAlO_2_), respectively. Four osteogenic inductive time points were 6, 9, 12, or 15 d, respectively. At the end of each induction time, the mineralization formation was detected and quantified. There were six replicates for each treatment.

In experiment 2, the siRNAs against DMP1 were designed, synthesized and transfected into primary cultured tibial osteoblasts of broiler chicks. Then, the inhibitory efficiencies of siRNAs against DMP1 were determined, and the most efficacious siRNA and optimal transfection time were selected to be used for subsequent experiments. There were a total of 5 treatments in a completely randomized design. The treatments included blank group (Blank), negative control siRNA group (NC), si340, si844, or si1028 group. Once the P1 cells grew to 60–80% confluence, transfection was performed according to the protocol. After transfection for 6 h, the transfection efficiency was measured by the flow cytometry. After 48 h transfection, the *DMP1* mRNA were determined by quantitative Real-Time PCR (RT-qPCR). Among the siRNAs we examined, si340 were the most efficient siRNA to be used for detecting the inhibitory efficiencies during the time points after transfection, and the time points were set at 12, 24, 48, 72, and 96 h, respectively. At the end of each time, *DMP1* mRNA were determined to calculate the inhibitory efficiencies of si340 against DMP1. There were six replicates for each treatment.

In experiment 3, it was conducted to investigate the effect of DMP1 silencing on tibial osteoblastic P retention rate, mineralization formation, alkaline phosphatase (ALP) activity, and bone gla-protein (BGP) content in primary cultured tibial osteoblasts of broiler chicks in order to address whether DMP1 was involved in regulating the bone P utilization of broilers using an *in vitro* model. A completely randomized design involving a 2 (cell types) × 3 (P supplemental levels) factorial arrangement of treatments was used in this experiment. The 2 cell types were control cells transfected with NC-siRNA and DMP1 silencing cells (transfected with si340). The 3 supplemental P levels were 0, 1, or 2 mmol L^−1^ of P as KH_2_PO_4_. Therefore, there were totally 6 treatments in this experiment. With or without si340 against DMP1, the P1 cells were incubated in OIM2 supplemented with 0.0, 1.0, or 2.0 mmol/L of P as NaH_2_PO_4_, respectively for 12 d. But note that DMEM in OIM2 were replaced by the P-free medium (DMEM with no phosphate, Gibco, Grand Island, NY, USA) in this experiment. The total P levels in the above mediums were 0.60, 1.65, and 2.65 mmol/L by analysis, respectively, and Ca level in each inductive medium used in the present study was 2.21 mmol/L by analysis. During the period of P treatment, transfection was performed every 3 days until samples needed to be collected. At the end of each incubation time, the cells were collected and prepared for determining P retention rate, mineralization formation, ALP activity, and BGP content. There were six replicates for each treatment.

### Detection and Quantification of Mineralization Formation

Detection and quantification of mineralization formation were determined as described previously (27). The cells were firstly washed with D-PBS twice and fixed with 4% paraformaldehyde for 15 min at room temperature. The fixed cells were then washed twice with D-PBS prior to the addition of 1 mL of 1% Alizarin Red S (ARS, pH 4.2) per well. For the quantification of ARS straining, 800 μL of 10% (v/v) acetic acid was added into each well and the plate was incubated in a shaker for 30 min at room temperature. Then, the cell monolayers were scraped from the plate and transferred with 10% (v/v) acetic acid to 1.5 mL tubes. After vortexing for 30 s, the slurry was overlaid with 500 μL mineral oil (Sigma-Aldrich, USA), heated to exactly 85°C for 10 min, and transferred to ice for 5 min. The slurry was then centrifuged at 20,000 g for 15 min, and the 500 μL of the supernatant was removed into a new 1.5 mL tube. To neutralize the acid, 200 μL of 10% (v/v) ammonium hydroxide were added to each sample. The absorbances of the ARS in aliquots (150 μL) of the supernatant were read at 405 nm in 96-well format using opaque-walled, transparent-bottomed plates (Corning, USA). Finally, the ARS concentrations were calculated according to the standard curve. In the present study, the mineralization formation was represented by the ARS concentration (mmol/L).

### Cell Transfection

Once the P1 cells grew to 60–80% confluence, transfection was performed according to the jetPRIME® RNAiMAX Transfection Reagent. Before transfection, the 4 μL of siRNA (20 μM) and 200 μL of jetPRIME® (Gibco-Invitrogen Corporation) were mixed and vortexed for 10 s, then supplemented with 4 μL jetPRIME® RNAiMAX transfection reagent, mixed for 1 s and incubated under room temperature for 10 min. Finally, 200 μL of the mixture were added to the cell containing well and mixed gently by rocking the plate back and forth. FAM-siRNA was used to confirm the transfection efficiency of siRNAs in primary cultured osteoblasts of broiler chicks. The siRNAs used in the present study were synthesized by the commercial company (GenePharma, Shanghai, China) and primer sequences are listed in Table 1.

**Table 1:** The siRNA primers used for the target and reference genes (experiments 2 and 3).

**Name**	**Sequences (5'-3')**
si340	Sense: GGAUAAGGAAGAGGGUGAATT Antisense: UUCACCCUCUUCCUUAUCCTT
si844	Sense: GGCAUCAGAUGAGGAGUCUTT Antisense: AGACUCCUCAUCUGAUGCCTT
si1028	Sense: CCUGACGAUGAUGCUCCAATT Antisense: UUGGAGCAUCAUCGUCAGGTT
NC-siRNA	Sense: UUCUCCGAACGUGUCACGUTT Antisense: ACGUGACACGUUCGGAGAATT
FAM-siRNA	Sense: FAM-UUCUCCGAACGUGUCACGUTT Antisense: FAM-ACGUGACACGUUCGGAGAATT

### Osteoblastic P Retention Rate

To determine the P retention rate of primary cultured tibial osteoblasts of broilers, the old medium was collected and pooled at every medium replacement. The total P contents in the fresh or old medium were determined by Inductively Coupled Plasma Optical Emission Spectrometry [5110 ICP-OES, Agilent Technologies Australia (M) Pty Ltd, Australia]. Accurately 1.0 mL of the fresh or old medium was taken in triplicate and digested with 12.5 mL of HNO_3_ and 2.5 mL of HClO_4_ at 200°C in a 50 mL calibrated flask until the solution became clear, and it was evaporated to almost dryness and diluted to about 15 mL with 2% HNO_3_ before analyses. Validation of the mineral analysis was conducted using soybean meal (GBW10013; National Institute of Standards and Technology, Beijing, China) as a standard reference material. The P retention rate of the tibial osteoblasts is defined as follows:


$$\begin{array}{l} \text{Tibial osteoblastic P}\\ \text{retention rate }\left(\text{\%}\right)=\frac{\text{V}1\times \text{C}1-\text{V}2\times \text{C}2}{\text{V}1\times \text{C}1}\times \;100, \end{array}$$

where V1 is total volume (mL) of the added fresh medium, C1 is total P content (mmol/L) in the fresh medium, V2 is total volume (mL) of the pooled old medium, and C2 is total P content (mmol/L) in the old medium.

### Determination of ALP Activity and BGP Content

The ALP activity was measured using a microplate reader with ALP assay kits (Nanjing Jiancheng Bioengineering Institute, Nanjing, China). The BGP content was determined by the method of ELISA with a commercial kit (Nanjing Jiancheng Bioengineering Institute, Nanjing, China) according to the manufacturer's instructions. The ALP activity and the BGP content in each sample were normalized by the protein concentration.

### RT-qPCR Analysis

The total RNA in the osteoblasts was isolated using TRIzol reagents (Life Technologies, Carlsbad, CA, USA) according to the manufacturer's protocols. Firstly, the cDNA synthesis was performed using PrimeScript RT Reagent Kit with cDNA Eraser (Qiagen, Chatsworth, CA, USA) according to the manufacturer's instructions. Subsequently, 50 ng of total cDNA was applied for RT-qPCR analysis using SYBR-Green PCR Master Mix (Life Technologies, Carlsbad, CA, USA). Finally, the relative mRNA expression of target gene was calculated using the 2^−ΔΔCt^ method, and the data were normalized by the geometric mean of two internal reference genes, β*-actin* and *GAPDH*. The primer sequences are listed in Table 2.

**Table 2:** Primers used for the target and reference genes (experiments 2 and 3).

**Genes**	**Sequence (5'−3')**	**Product length (bp)**	**GenBank ID**
*DMP1*	Forward: GCCTGACGATGATGCTCCAA Reverse: TGGATGTGCTCTCTTCGCTC	116	NM_206993.1
*β-actin*	Forward: ACCTGAGCGCAAGTACTCTGTCT Reverse: CATCGTACTCCTGCTTGCTGAT	169	NM_205518.1
*GAPDH*	Forward: CTTTGGCATTGTGGAGGGTC Reverse: ACGCTGGGATGATGTTCTGG	82	NM_204305.1

*DMP1, dentin matrix protein 1; GAPDH, glyceraldehyde-3-phosphate dehydrogenase*.

### Western Blotting

At the end of the P treatments, whole cell lysates were harvested in RIPA buffer (Beyotime, Shanghai, China). Cell lysates containing 10 μg of each protein were then subjected to a 10% SDS-PAGE, and transferred onto a polyvinylidene difluoride membrane (Merck Millipore, Billerica, MA, USA). After blocking with 5% bovine serum albumin, the membrane was incubated overnight at 4°C with primary antibody against DMP1 (Abcam, Cambridge, UK) or β-tubulin (Huaxingbio, Beijing, China). After washing with Tris-buffered saline containing 0.02% (v/v) Tween-20 (TBST) three times for 5 min each, the membranes were incubated with the secondary antibody of goat anti-rabbit (Huaxingbio, Beijing, China) or goat anti-mouse (Huaxingbio, Beijing, China). After washing with TBST three times for 5 min each, protein bands were visualized using enhanced chemiluminescence substrate (Tanon, Shanghai, China) and quantified with an ImageQuant LAS4000 scanner (GE Healthcare Life Sciences, Pittsburgh, PA), followed by analysis with TotalLab Quant Software (TotalLab, Newcastle on Tyne, UK). The β-tubulin protein was used to normalize the expression levels of target proteins.

### Statistical Analyses

Statistical analyses were performed by one-way ANOVA for the data from experiment 1 and experiment 2, and by two-way ANOVA for the data from experiment 3 using the GLM procedure of SAS 9.4 (SAS Institute Inc., Cary, NC). The model for experiment 3 included the main effects of cell type, P supplemental level and their interaction. Orthogonal polynomials were applied for testing linear and quadratic effects of dependent variables to independent variables for the data from experiments 1 and 2. The LSD method was used to test the differences between treatment means. Each replicate served as an experimental unit. Significant differences were set at *P* ≤ 0.05.

## Results

### Screening of the Optimal Inductive Medium and Time (Experiment 1)

**Figure 1:**
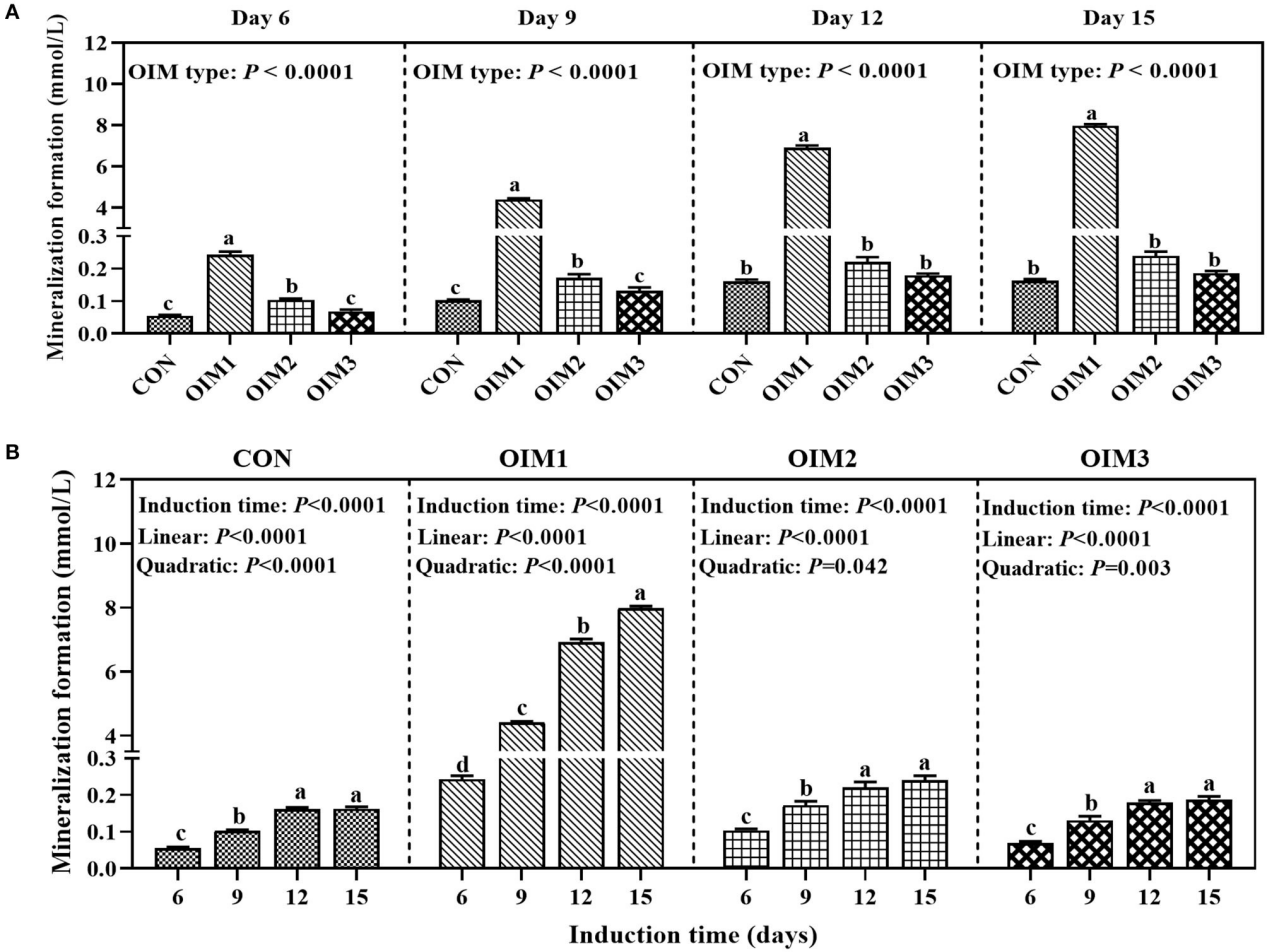
Effect of OIM type **(A)** or induction time **(B)** on mineralization formation in primary cultured tibial osteoblasts of broiler chicks (experiment 1). All values are expressed as means ± SE, *n* = 6. Means without a common letter differ, *P* < 0.05. CON, complete culture medium; OIM1, complete culture medium supplemented with 100 nM Dex, 50ug/mL Asc, and 10 mmol/L β-Gly; OIM2, complete culture medium supplemented with 100 nM Dex and 50ug/mL Asc; OIM3, OIM2 supplemented with 10 mmol/L NaAlO_2_.

As shown in Figure 1A, the OIM type had a significant effect (*P* < 0.0001) on the mineralization formation in tibial osteoblasts of broilers at 6, 9, 12, and 15 d. At 6, 9, 12, and 15 d of induction, osteoblastic mineralization in OIM1 group was significantly higher (*P* < 0.0001) than those in CON, OIM2 and OIM3 groups. At 6 and 9 d of induction, mineralization formation in OIM2 group was significantly higher (*P* < 0.045) than those in CON and OIM3 groups, however, there was no significant difference (*P* > 0.121) between CON and OIM3 groups. At 12 and 15 d of induction, there was no significant difference (*P* > 0.117) in mineralization formation between CON, OIM2, and OIM3 groups. The OIM2 without β-Gly was selected for the following experiment since the β-Gly in OIM1 contains P which would interfere with the effects of added P levels.

As shown in Figure 1B, inductive time had a significant effect (*P* < 0.0001) on osteoblastic mineralization formation under all OIM culture conditions. The mineralization formation increased linearly (*P* < 0.0001) and quadratically (*P* ≤ 0.042) with the increase of inductive time, and remained at a stabilized level from 12 to 15 d of incubation. Therefore, we selected 12 d as P treatment time in experiment 3.

### Detection of siRNA Transfection Efficiencies by Flow Cytometry (Experiment 2)

**Figure 2:**
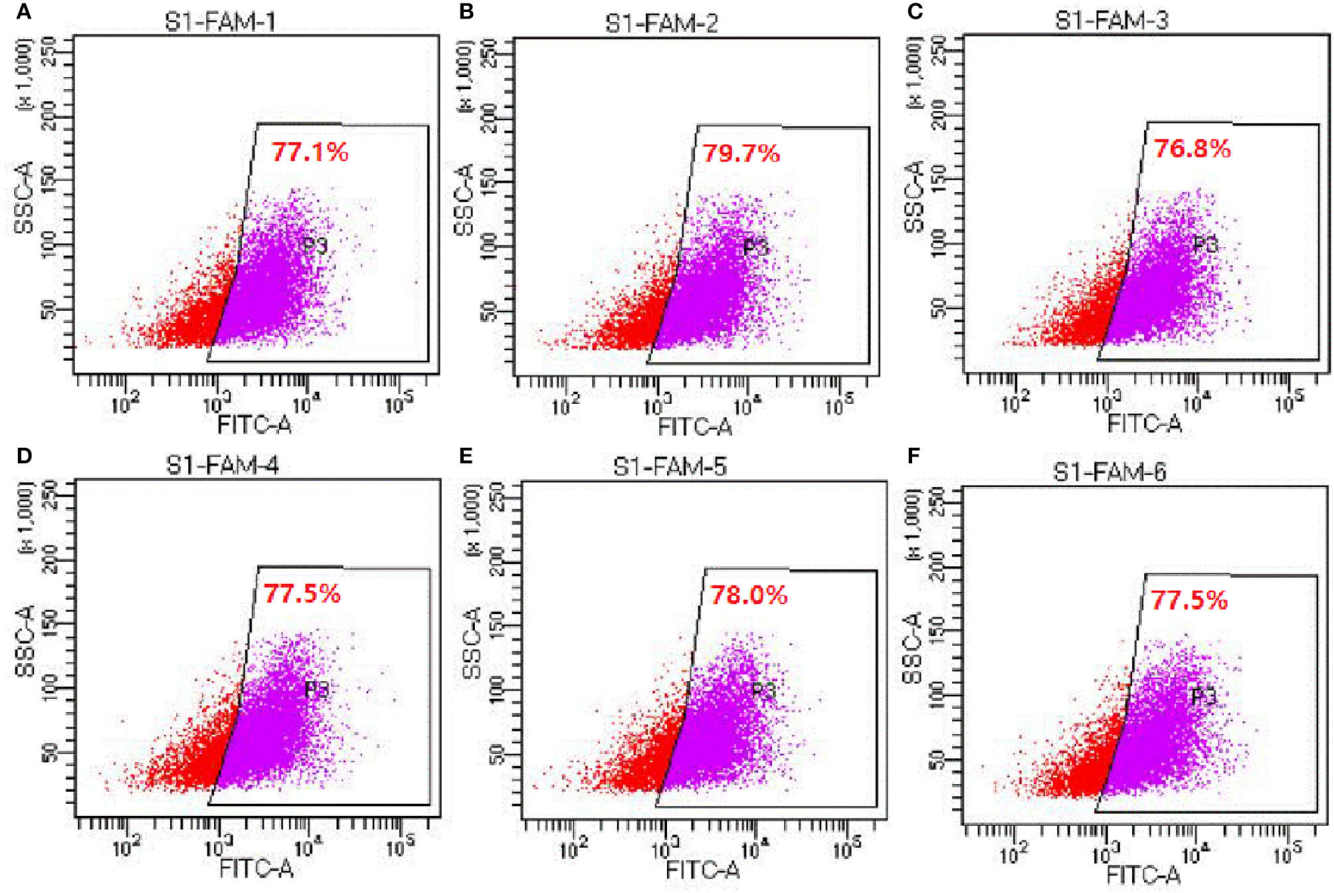
Detection of siRNA transfection efficiency by flow cytometry (experiment 2). **(A–F)** Represents the six replicates, respectively, *n* = 6.

Six hours after transfection, the transfection efficiency was measured by flow cytometry. The results showed that the transfection efficiencies reached to 76.8–79.7% (Figure 2), indicating that siRNA had been successfully transfected into cells.

### Inhibitory Efficiencies of SiRNAs and Transfection Time (Experiment 2)

**Figure 3:**
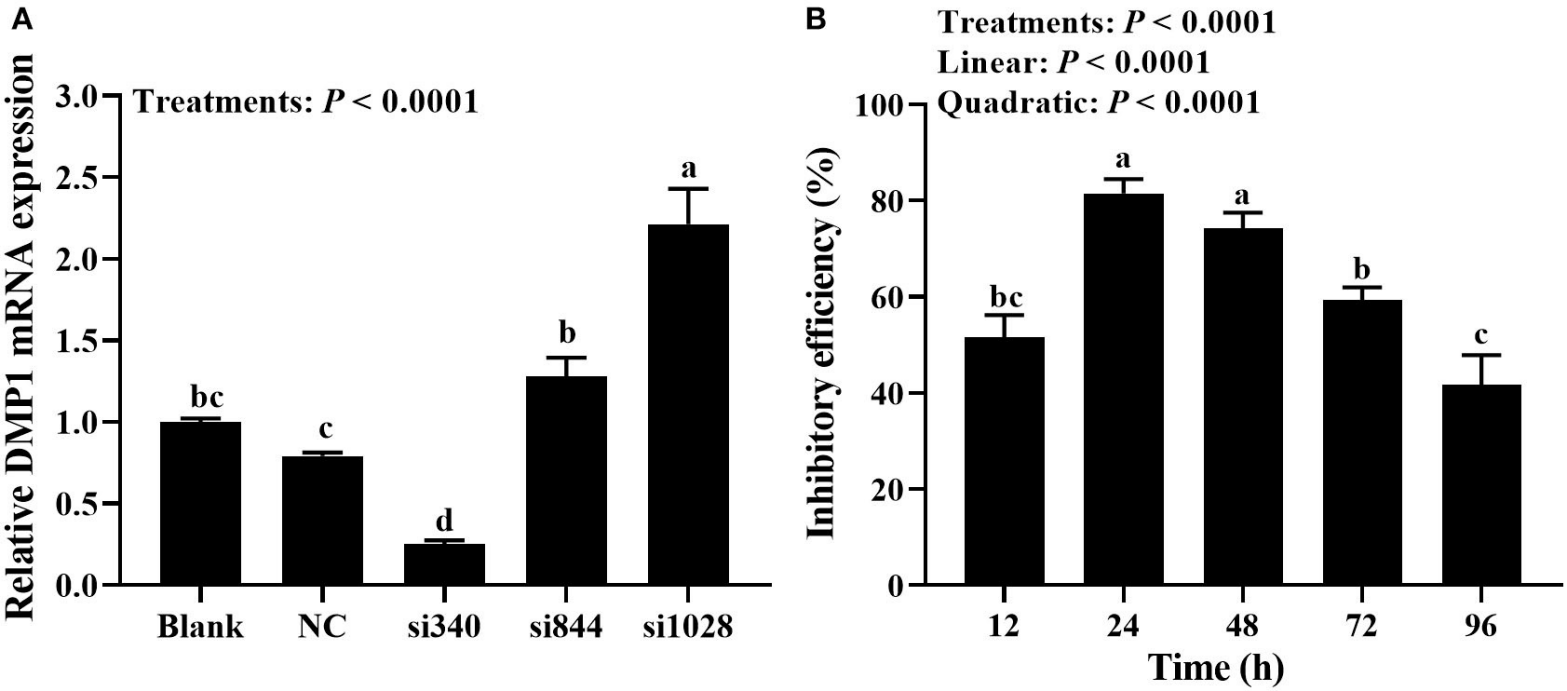
Detection of transfection efficiency and selection of next time point for transfection (experiment 2). **(A)** Screening of the most effective siRNAs against *DMP1* mRNA; **(B)** Effective time of si340 against *DMP1* mRNA. Relative mRNA expression of DMP1 was calculated using the 2^−ΔΔCt^ method, and the data were normalized by the geometric mean of two internal reference genes β*-actin* and *GAPDH*. All values are expressed as means ± SE, *n* = 6. Means without a common letter differ, *P* < 0.05. *DMP1*, dentin matrix protein 1.

Primary cultured tibial osteoblasts of broiler chicks were transfected with DMP1 specific siRNAs (namely si340, si844, and si1028) or their control (NC-siRNA). After 48 h, cells were collected for RT-qPCR assay. As shown in Figure 3A, treatments affected (*P* < 0.0001) *DMP1* mRNA expression of broiler tibial osteoblasts. Compared with blank and NC, the si340 decreased (*P* < 0.05) *DMP1* mRNA expression, but the si1028 increased (*P* < 0.05) it. The expression of *DMP1* mRNA in si340 group was significantly lower (*P* < 0.0001) than those in si844 and si1028 groups. There were no differences (*P* > 0.087) in *DMP1* mRNA expression among blank, si844 and NC groups. The expression of *DMP1* mRNA in the si844 group was significantly lower (*P* < 0.0001) than that in the si1028 group, but higher (*P* = 0.005) than that in the NC group.

As shown in Figure 3B, the inhibitory efficiency of *DMP1* mRNA was affected (*P* < 0.0001) by transfection time of si340. The inhibitory efficiency of *DMP1* mRNA increased linearly and quadratically (*P* < 0.0001) with the increasing transfection time of si340. The inhibitory efficiency of the si340 on *DMP1* mRNA was 81.5% at 24 h after transfection, which was significantly higher (*P* ≤ 0.001) than those at 12, 72 and 96 h, but no significantly different (*P* = 0.229) from that at 48 h. The inhibitory efficiency of the si340 on *DMP1* mRNA was significantly lower (*P* ≤ 0.016) at 72 h than those at 24 and 48 h, but not significantly different (*P* = 0.203) from that at 12 h, and significantly higher (*P* = 0.006) than that at 96 h.

### Construction of the DMP1 Silencing Model of Osteoblasts Under Different P Levels (Experiment 3)

**Figure 4:**
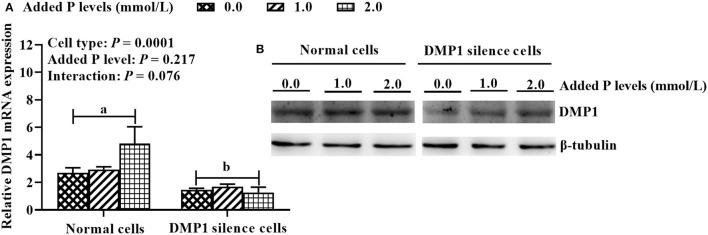
*DMP1* silencing model for primary cultured tibial osteoblasts of broiler chicks under different P levels (experiment 3). **(A)** *DMP1* mRNA expression; **(B)** DMP1 protein expression. Relative mRNA expression of *DMP1* was calculated using the 2^−ΔΔCt^ method, and the data were normalized by the geometric mean of two internal reference genes β*-actin* and *GAPDH*. The β-tubulin protein was used to normalize the expression level of target protein. All values are expressed as means ± SE, *n* = 6. Means without a common letter differ, *P* < 0.05. *DMP1*, dentin matrix protein 1.

As shown in Figure 4, cell type had a significant effect (*P* = 0.0001) on the expression of *DMP1* mRNA in osteoblasts, while P level and their interaction had no significant effects (*P* > 0.075). Compared with normal cells, DMP1-si340 transfection significantly reduced (*P* = 0.0001) the expression of *DMP1* mRNA in osteoblasts, and inhibition efficiency was 57.8%. In addition, DMP1 protein expression was also clearly inhibited.

### Effect of DMP1 Silencing on P retention Rate, Mineralization Formation, ALP Activity and BGP Content in Primary Cultured Tibial Osteoblasts of Broiler Chicks (Experiment 3)

**Figure 5:**
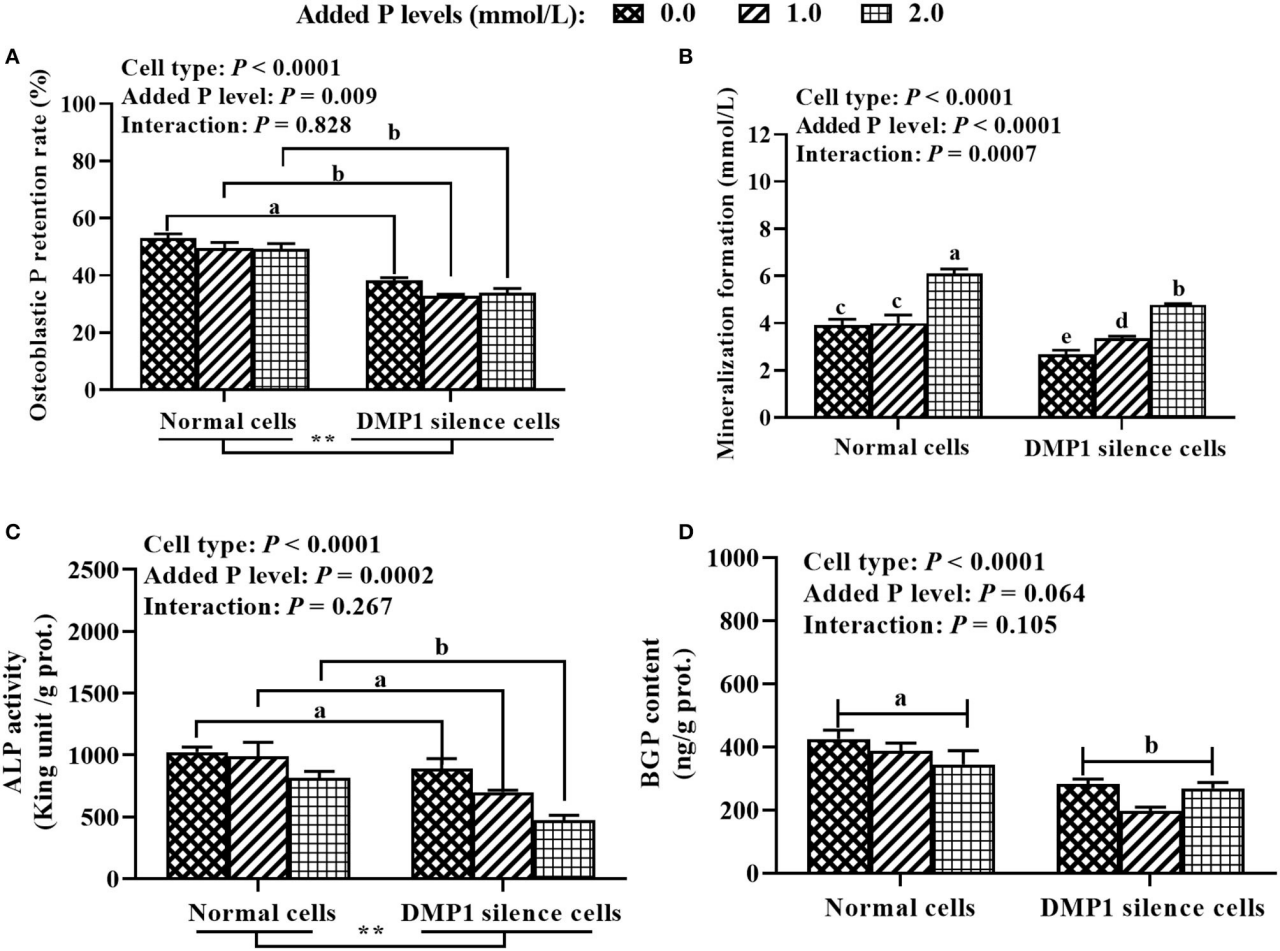
Effect of DMP1 silencing on tibial osteoblastic P retention rate **(A)**, mineralization formation **(B)**, ALP activity **(C)**, and BGP content **(D)** in primary cultured tibial osteoblasts of broiler chicks (experiment 3). All values are expressed as means ± SE, *n* = 6. Means without a common letter differ, *P* < 0.05. ** *P* < 0.01. DMP1, dentin matrix protein 1; ALP, alkaline phosphatase; BGP, bone gla protein.

As shown in Figure 5, the results showed that cell type had an effect (*P* < 0.0001) on P retention rate, ALP activity and BGP content in osteoblasts. The added P level had an effect (*P* ≤ 0.009) on P retention rate and ALP activity in osteoblasts. The cell type × added P level interaction had an effect (*P* = 0.0007) on osteoblastic mineralization formation. The DMP1 silencing decreased (*P* ≤ 0.009) P retention rate, ALP activity and BGP content in osteoblasts. The tibial osteoblastic P retention rate was higher (*P* < 0.01) at 0.0 mmol/ L of added P than those at 1.0 and 2.0 mmol/L, and no difference (*P* = 0.78) was observed between 1.0 and 2.0 mmol/L of added P. At any of added P levels, DMP1 silencing decreased (*P* < 0.0001) the osteoblastic mineralization formation, however, its greatest decrease by DMP1 silencing was observed at the highest added P level (2.0 mmol/L). Osteoblastic ALP activity was lower (*P* < 0.005) at 2.0 mmol/L of added P than those at 0.0 and 1.0 mmol/L, with no difference (*P* = 0.095) between 0.0 and 1.0 mmol/L of added P.

## Discussion

The hypothesis that DMP1 silencing would inhibit P utilization, and thus DMP1 would be involved in regulating P metabolic utilization in primary cultured tibial osteoblasts of broiler chicks has been supported by the results of the present study. In the present study, DMP1 silencing decreased the expressions of *DMP1* mRNA and protein, P retention rate, mineralization formation, ALP activity and BGP content in osteoblasts, indicating that DMP1 silencing inhibited P utilization, and thus DMP1 was involved in regulating P metabolic utilization in primary cultured tibial osteoblasts of broiler chicks. The above new findings have been not reported before, and provide a novel insight into the regulation of the P utilization in the bone of broilers, and will contribute to develop feasible strategies to improve the bone P utilization efficiency of broilers so as to decrease its excretion.

The optimal OIM type and inductive time for promoting the osteoblastic mineralization were screened according to the capacity of the osteoblastic mineralization. The results of the present study showed that the inductive effects of four OIMs on the osteoblastic mineralization were as follows: OIM1 > OIM2 > OIM3 > CON. However, the most effective OIM1 contained β-Gly. During mineralization *in vitro*, the β-Gly serves as a P source for mineralization (28), and its presence would interfere with the effects of addded P levels. Therefore, the OIM2 without β-Gly was selected for the subsequent experiment.

At least a 3-weeky period of continuous treatment of a confluent monolayer of cells with Dex, in combination with Asc and β-Gly, is required for osteogenic differentiation (28). According to a previous study, the remarkable increases of mineralized nodules and areas were observed from day 7 to 21 d after induction of mineralization in MC3T3-E1 cells (29). However, osteoblastic mineralization cycle is still controversial from 7 to 21 d after adding mineralization medium (30–32). It is reported that the osteoblasts isolated from rat tibia formed mineralized nodules after 41 d (33). Other studies also demonstrated that the osteoblasts from rat calvaria bones were cultured for 7, 14, 21 and 28 d, and the extent of mineralization increased continuously (34, 35). We previously showed that the number and area of mineralized nodules increased linearly and quadratically with the increase of incubation time, and tended to stabilize from 24 to 32 d of incubation (14). The above disparities might be majorly due to the differences in the cell types of different species. Our results showed that the optimal inductive time was 12 d. These above findings provided scientific experimental bases for the follow-up study of regulating P utilization in primary tibial osteoblasts of broiler chicks.

Small interfering RNA mediated gene silencing is one of the most commonly-used methods to study the gene functions because of its target specificity, potency and ability to silence the expression of virtually any gene (36). Primary cells are notoriously difficult to transfect and susceptible to the toxic effects of transfection reagents. A transfection reagent with a high transfection efficiency and low cytotoxicity at shorter periods of time following transfection when cytotoxicity was limited, allowing sufficient yield of transfected primary cells for use in subsequent assays. In the present study, the si340 could effectively inhibit the expression of *DMP1* mRNA in tibial osteoblasts, and the inhibitory efficiency reached 74% as detected after 48 h. Furthermore, the inhibition of si340 against DMP1 was transient, which meant that si340 did not keep DMP1 expression low all time on long osteogenic culture. With inhibitory efficiency >50% as the reference value, the duration of the effective inhibition of *DMP1* mRNA expression by si340 was 72 h, which could be used as the time point of the next transfection of si340 in subsequent experiments.

The DMP1 is a bone matrix protein specifically and highly expressed in osteoblasts, hypertrophic chondrocytes and osteocytes as well as pre-osteocytes (15). The DMP1 has multiple roles including direct effects on mineral formation and mineral crystal growth, whereas other characteristics are secondary to impaired P homeostasis (37, 38). The abnormalities observed in DMP1-null mice included defective osteocyte maturation and increased FGF23 expression, leading to pathological changes in bone mineralization (19, 21). To clarify the role of DMP1 in regulating P utilization of broiler osteoblasts, we successfully established DMP1 silencing model in the present study. And then, we found that DMP1 silencing decreased osteoblastic P retention rate, mineralization formation, ALP activity and BGP content. It is well-known that matrix mineralization plays a critical role in bone formation. At the end of osteogenic differentiation, mineralized nodules formed in osteoblasts. Bone mineralization is a complex process modulated by organic macromolecules under cellular control (7). Bone ALP, BGP, and DMP1 are the important regulators of bone matrix mineralization and adjust the hydroxyapatite formation (39–41). The P is an essential mineral to form hydroxyapatite for bone mineralization, and imbalance of P could inhibit osteoblastic mineralization (42). A series of studies showed that bone ash, P retention, bone mineral content and density were affected by dietary P level, and increased linearly or quadratically as dietary P levels increased (1–3, 43). The ALP is one of the most frequently-used biochemical marker of osteoblastic activity (44), and it can hydrolyze phosphate ester to provide the P for promoting the hydroxyapatite formation (39). It was reported that ALP is expressed in the early stage of osteogenic differentiation, and BGP is necessary for osteogenic differentiation at the late stage (45). In the present study, the added P level decreased P retention rate and ALP activity, but increased mineralization formation, while a decreased trend was noted for BGP content. Our previous studies also demonstrated that tibia ALP activity and BGP content decreased with the increase of age and dietary non-phytate phosphorus level (1, 46), which agrees with the above *in vitro* results of the present study. These aforementioned results demonstrate that DMP1 is indeed involved in regulating P metabolic utilization in broiler osteoblasts, and it would be an important regulator in promoting the P utilization in the bone of broilers. However, the detailed molecular mechanisms underlying the transcriptional and translational regulations of DMP1 gene expression in promoting osteoblastic P utilization need to be further studied in the future.

## Conclusions

The results from the present study indicated that DMP1 silencing inhibited P utilization, and thus DMP1 was involved in regulating P metabolic utilization in primary cultured tibial osteoblasts of broiler chicks.

## Data Availability Statement

The original contributions presented in the study are included in the article/supplementary material, further inquiries can be directed to the corresponding author/s.

## Author Contributions

TL: data curation and writing—original draft preparation. YG: investigation and formal analysis. YH and XC: resources. WZ and LZ: investigation. FG: methodology. ZL: review & correction. XL: supervision and writing—review & editing. All authors contributed to the article and approved the submitted version.

## Funding

The present study was financially supported by the Key Program of the National Natural Science Foundation of China (Project no. 31630073; Yangzhou, P. R. China), the Natural Science Foundation of Jiangsu Province (Project no. BK20210809; Yangzhou, P. R. China), and the Initiation Funds of Yangzhou University for Distinguished Scientists (Yangzhou, China).

## Conflict of Interest

The authors declare that the research was conducted in the absence of any commercial or financial relationships that could be construed as a potential conflict of interest. The handling Editor declared a past co-authorship with several of the authors XL and LZ.

## Publisher's Note

All claims expressed in this article are solely those of the authors and do not necessarily represent those of their affiliated organizations, or those of the publisher, the editors and the reviewers. Any product that may be evaluated in this article, or claim that may be made by its manufacturer, is not guaranteed or endorsed by the publisher.
